# TprA/PhrA Quorum Sensing System Has a Major Effect on Pneumococcal Survival in Respiratory Tract and Blood, and Its Activity Is Controlled by CcpA and GlnR

**DOI:** 10.3389/fcimb.2019.00326

**Published:** 2019-09-13

**Authors:** Anfal Shakir Motib, Firas A. Y. Al-Bayati, Irfan Manzoor, Sulman Shafeeq, Anagha Kadam, Oscar P. Kuipers, N. Luisa Hiller, Peter W. Andrew, Hasan Yesilkaya

**Affiliations:** ^1^Department of Infection, Immunity & Inflammation, University of Leicester, Leicester, United Kingdom; ^2^Department of Microbiology, College of Medicine, University of Diyala, Baqubah, Iraq; ^3^College of Pharmacy, University of Kirkuk, Kirkuk, Iraq; ^4^Molecular Genetics, University of Groningen, Groningen, Netherlands; ^5^Department of Biological Sciences, Carnegie Mellon University, Pittsburgh, PA, United States

**Keywords:** *Streptococcus pneumoniae*, transcriptional regulation, TprA/PhrA, sugar metabolism, virulence

## Abstract

*Streptococcus pneumoniae* is able to cause deadly diseases by infecting different tissues, each with distinct environmental and nutritional compositions. We hypothesize that the adaptive capabilities of the microbe is an important facet of pneumococcal survival in fluctuating host environments. Quorum-sensing (QS) mechanisms are pivotal for microbial host adaptation. We previously demonstrated that the TprA/PhrA QS system is required for pneumococcal utilization of galactose and mannose, neuraminidase activity, and virulence. We also showed that the system can be modulated by using linear molecularly imprinted polymers. Due to being a drugable target, we further studied the operation of this QS system in *S. pneumoniae*. We found that TprA controls the expression of nine different operons on galactose and mannose. Our data revealed that TprA expression is modulated by a complex regulatory network, where the master regulators CcpA and GlnR are involved in a sugar dependent manner. Mutants in the TprA/PhrA system are highly attenuated in their survival in nasopharynx and lungs after intranasal infection, and growth in blood after intravenous infection.

## Introduction

*Streptococcus pneumoniae* causes an array of diseases with high morbidity and mortality including pneumonia, bacteraemia, meningitis, and otitis media (Weiser et al., 2018), especially among children and elderly. The high incidence of pneumococcal diseases is compounded by a rising trend of antimicrobial resistance among pneumococcal strains. This requires new approaches to develop anti-infectives against this microbe. Adaptation to changing environmental conditions has been linked to pneumococcal virulence (Sandrini et al., 2014; Kadam et al., 2017; Zhi et al., 2018) hence, pneumococcal proteins involved in detection and processing of environmental signals may be targeted to develop new anti-infectives.

Upon detection of environmental alterations, bacteria adjust their gene expression in order to obtain a phenotype that is suitable for the environment to which they are exposed (Federle, 2012). Transcriptional regulators enable the microbes to sense and respond to environmental stimuli, by altering gene expression. While individual cells have inherent adaptive capability, in certain cases, the adaptation to a given stimulus occurs at the population level via detection and processing of chemical signals. Community level coordination of gene expression is known as quorum sensing (QS). QS systems have been implicated in wide range of biological functions, including surface attachment, oxidative stress resistance, microbial competition, and virulence (Federle, 2012; Cuevas et al., 2017; Zhi et al., 2018). These systems have received considerable attention not least because of their far-reaching impact on the fundamental biology of microbes but also from the more applied perspective for their utility as anti-infective targets (Brooks and Brooks, 2014).

In the pneumococcus, the competence (*com*) locus and LuxS-mediated AI-2 are the best-studied QS systems (Alloing et al., 1996; Trappetti et al., 2017). However, functional genomic studies have revealed several other QS systems in *S. pneumoniae* (Hoover et al., 2015; Cuevas et al., 2017; Junges et al., 2017; Kadam et al., 2017; Motib et al., 2017; Aggarwal et al., 2018; Zhi et al., 2018). Prominent among these is the RRNPP (Rap, Rgg, NprR, PlcR, PrgX) family of transcriptional regulators, which are characterized at the structural level by the presence of tetratricopeptide repeats that mediate regulator-peptide interactions (Cook and Federle, 2014). Recently, we and others characterized several Rgg (Rgg144, Rgg939) and TprA (TprA, TprA2) family regulators in *S. pneumoniae* (Hoover et al., 2015; Cuevas et al., 2017; Junges et al., 2017; Kadam et al., 2017; Motib et al., 2017; Zhi et al., 2018). We found that Rgg144 and Rgg939 QS circuits are induced by short hydrophobic peptides (Shp) upon detection of galactose or mannose (Zhi et al., 2018). Microarray analyses revealed that the expression of a large number of genes involved in essential physiological functions and virulence is controlled by Rgg/Shp QS systems. Interestingly, our data revealed evidence for cross-talk between these systems, and showed the involvement of Rgg systems in colonization and virulence (Zhi et al., 2018). Additionally, we studied a novel streptococcal cell-cell communication peptide, known as virulence peptide 1 (VP1), regulated by Rgg/Shp144, which promotes pneumococcal virulence and biofilm formation (Cuevas et al., 2017).

TprA/PhrA is another member of the RRNPP family found in pneumococci. Hoover et al. showed PhrA/TprA QS system's role in galactose metabolism and the modulation of a predicted lantibiotic gene cluster (Hoover et al., 2015). TprA is a PlcR ortholog (Hoover et al., 2015), first characterized in *Bacillus*, and reported to control expression of genes for most of their extracellular virulence factors (Agaisse et al., 1999; Gohar et al., 2008). The function of this regulator relies on the existence of a signaling peptide (PapR), the gene of which is located about 70 bp downstream from *plcR* (Gominet et al., 2001). Once imported, PapR binds PlcR, modifying its interaction with the PlcR box, located in the promoter regions of PlcR regulated genes (Rocha-Estrada et al., 2010), and controlling their transcription. The number of TprA/PhrA homologs varies across pneumococcal lineages (Kadam et al., 2017). Specifically, we have shown that strains in the PMEN1 lineage (also known as Spain23F-1 and SPN23F) encode not only TprA/PhrA, but also the homologous TprA2/PhrA2 signaling systems. We showed that TprA2/PhrA2 is a negative regulator of a genomic locus encoding a lanthionine-containing peptide (*lcpA*), and the presence of this QS system restrains the virulence of PMEN1 through the control exerted over *lcpA* expression. Interestingly, PhrA2 can induce the expression of the TprA/PhrA system not only in PMEN1 strains, but also in type 2 strain D39 from a divergent lineage. This suggests that this feature may allow PMEN1 strains to modify the transcriptomic and functional attributes of a multi-strain pneumococcal community. Given that an estimated 20–40% of pneumococcal infections are multi-strains, the implication of this finding is considerable for understanding pneumococcal virulence and anti-infective design (Everett et al., 2012; Saha et al., 2015).

While the TprA2/PhrA2 system restrains virulence in a mouse colonization model, the TprA/PhrA strain is essential for virulence in the type 2 D39 strain, as we demonstrated in our recent study using the models of mouse pneumococcal pneumonia and chinchilla otitis media (Kadam et al., 2017; Motib et al., 2017). It was shown by us, and others, that PhrA levels are repressed in glucose and raised in galactose and that the TprA/PhrA system controls the expression of a predicted lantibiotic gene cluster that encodes members of the bacteriocin family of antimicrobial peptides (Hoover et al., 2015). Our data also indicated that TprA/PhrA controls a neuraminidase gene expression and synthesis when the pneumococcus is grown on galactose (Motib et al., 2017). Moreover, we were able to modulate the function of TprA/PhrA system using linear molecularly imprinted polymers (LMIP), which interferes with the interaction of TprA/PhrA, thus, abrogating phenotypic manifestation of the system both *in vitro* and *in vivo* (Motib et al., 2017). This clearly showed that TprA/PhrA is a drugable anti-infective target. Therefore, to extend our knowledge of this QS system, we studied the regulatory interactions between TprA and PhrA, and assigned a role to CcpA and GlnR in regulation of TprA/PhrA. In addition, we have further evaluated TprA/PhrA's role in pneumococcal growth in the nasopharynx and lungs, and in septicaemia.

## Materials and Methods

### Bacterial Strains and Growth Conditions

The strains used in this study are listed in Table S1. *S. pneumoniae* D39 was grown microaerobically at 37°C either in brain heart infusion broth (BHI), blood agar base (BAB) (Oxoid, UK) supplemented with 5% (v/v) defibrinated horse blood, or in chemically defined medium (CDM) supplemented with 55 mM of the selected sugar (Yesilkaya et al., 2006, 2008). For anaerobic growth, an anaerobic cabinet was used. *Escherichia coli* was grown in Luria broth with shaking at 37°C, or in Luria agar (Oxoid). Growth was monitored by measuring the OD600, or by determining colony counts. Growth rates (μ) were calculated through linear regressions of the plots of ln(OD600) vs. time during the exponential growth phase. Spectinomycin, tetracycline, and kanamycin were added at 100, 15, and 250 μg/mL, respectively, for pneumococcal cultures, and for *E. coli* ampicillin and kanamycin were used at 100 and 50 μg/mL, respectively.

### Construction of Genetically Modified Strains

Insertion-deletion mutants were constructed by transforming an *in vitro* mutagenized SOEing construct as described before (Al-Bayati et al., 2017). Briefly, a spectinomycin (Spec^R^) resistance gene cassette was amplified using the primes Spec-F and Spec-R (Table S2). The primers LF-SOEX-F/LF-SOEX-R and RF-SOEX-F/RF-SOEX-R (where X indicates the gene code) were used to amplify the left and right flanking regions of each target gene, generating PCR products of ~600 bp. The amplified antibiotic resistance cassette and the PCR products flanking the target gene were then fused using LF-SOEX-F and RF-SOEX-R primers. The fused product was gel-purified after electrophoresis (Qiagen) and transformed into *S. pneumoniae* (Bricker and Camilli, 1999). The transformants were selected on BAB plates containing the appropriate antibiotic and the mutations were confirmed by PCR and sequencing. To rule out the polar effect of mutation, an intact copy of *tprA* with its putative promoter was introduced into Δ*tprA* using the pCEP plasmid (Guiral et al., 2006; Table S1).

Chromosomal transcriptional *lacZ* fusions to the target promoters were constructed by using the integration plasmid pPP1 (Halfmann et al., 2007). The putative promoter regions were amplified using the primers modified to incorporate *SphI* and *BamHI* sites (indicated with Fusion-F/R in Table S2, and then ligated into similarly digested pPP1. All plasmid constructs were confirmed by sequencing, and integrated into pneumococcal genome via double crossover. β-galactosidase activity was measured as described before using cells grown anaerobically in CDM at 37°C supplemented with 55 mM of the selected sugars (Kadam et al., 2017; Motib et al., 2017).

### RNA Extraction From Bacterial Cells, cDNA Synthesis, and Quantitative RT-PCR

The Trizol reagent kit (Invitrogen, UK) was used to isolate the total RNA. First strand cDNA synthesis was done at 42°C for 50 min using SuperScript III reverse transcriptase and random hexamers, according to the manufacturer's instructions (Invitrogen, UK). cDNA (15 ng) was amplified in a 20 μL reaction volume containing 1X SensiMix SYBR Master mix (Bioline, UK) and 3 pmol of each primer (Table S2). The transcription level of specific genes was normalized to *gyrB* transcription, which was amplified in parallel with SPD0709F and SP0709R primers. The results were analyzed by the comparative C_T_ method (Livak and Schmittgen, 2001).

### Microarray Analysis

*Streptococcus pneumoniae* D39 and its isogenic mutant strain Δ*tprA* were grown microaerobically in CDM supplemented either with 55 mM galactose or mannose as the main carbon source (Gaspar et al., 2014), or in M17 medium supplemented with 0.5% glucose (Difco) The experiments were repeated with four biological replicates. The MicroPrep software package was used to obtain the microarray data from the slides. CyberT implementation of a variant of the *t*-test (http://wiki.gcc.rug.nl/wiki/GccStart) was performed and false discovery rates (FDRs) were calculated (Gominet et al., 2001). For differentially expressed genes, *p* < 0.001 and FDR < 0.05 were taken as standard. For the identification of differentially expressed genes, a Bayesian *p*-value of < 0.001 and a fold change cut-off 2-fold was applied. All other procedures for the DNA microarray experiments and data analysis were performed as described before (Shafeeq et al., 2015). Microarray data have been submitted to GEO (Gene Expression Omnibus) database under the accession numbers GSE 67668 and GSE133010.

### Cloning and Expression of Recombinant Proteins

*ccpA, tprA*, and *glnR* coding regions were amplified by PCR, and the amplicons were cloned into pLEICS-01 (Al-Bayati et al., 2017). The recombinant plasmid was transformed into *E. coli* BL21 (DE3) and gene expression was induced with 0.5 mM IPTG at 25°C. Recombinant proteins were then purified using Talon® Metal Affinity resin (Clontech Inc., UK) as previously described (Zhi et al., 2018). The identity of the purified recombinant proteins was achieved by matrix-assisted laser desorption ionization—time of flight (MALDI-TOF) done by PNACL (University of Leicester).

### Enzyme Assays

Neuraminidase activity was assayed using chromogenic 2-O-(p-nitrophenyl) -α-D-*N*-acetylneuraminic acid (pNP- NANA) (Sigma, United Kingdom), as previously described (Yesilkaya et al., 2006). The absorbance of released p-nitrophenol was measured photometrically at 405 nm. The protein concentration was measured according to the method described by Bradford (1976). One unit of enzyme activity was defined as the amount of enzyme that produced 1 μmol p-nitrophenol per min per milligram of protein under standard assay conditions.

### Electrophoretic Mobility Shift Assay (EMSA)

Putative promoter regions and the presence of regulatory elements of target genes were identified using the bacterial promoter prediction tool (BPROM) (Solovyev et al., 2010) and the motif-based sequence analysis tools (MEME). The putative promoter regions were amplified using EMSA-F/R primers (Table S2).

The binding reaction was set up by mixing a constant amount of target promoter probes (~10 nmol), and increasing amounts (0.1–0.5 μM) of purified and dialyzed His-tagged proteins, in the presence of 5X binding buffer (20 mM Tris-HCl pH 7.5, 30 mM KCl, 1 mM DTT, 1 mM EDTA pH 8.0 and 10% v/v glycerol) at room temperature for 20 min in a total volume of 20 μL. The binding reaction mixture was run on an 8% w/v non-denaturing polyacrylamide gel. The detection of DNA-protein complexes relied on SYBR green staining (Molecular Probes fluorescence-based EMSA kit, Invitrogen), or the use of 5′-FAM labeled DNA probe, obtained with primers that incorporate fluorescently labeled tags. Typhoon Trio^+^ scanner (GE Healthcare Life Sciences) with a 526 nm short-pass wavelength filter was used for visualizing DNA-protein complexes.

### *In vivo* Studies

Female, 8–10 week old MF1 mice weighing ~30–35 g (Charles River, UK), were anesthetized with 2.5% v/v isoflurane (Astra Zeneca, Macclesfield, UK) over oxygen (2–2.5 liter/min). For the pneumonia model, ~1.5 × 10^6^ pneumococci, in 50 μL PBS (Gaspar et al., 2014; Glanville et al., 2018) were administered dropwise into the nostrils of the anesthetized mouse, held vertically. After infections, CFU were determined to ensure that the intended number of pneumococci had been administered. At 24 and 48 h post-infection, a pre-selected group of mice was culled under terminal anesthesia. Nasopharyngeal tissues and the lungs were removed, homogenized, and pneumococcal numbers determined by plating out the serial dilutions of the homogenates.

For intravenous infection, mice were infected with ~1 × 10^6^ CFU in 100 μL PBS, pH 7.0, through a dorsal tail vein. Mice were observed for disease signs over 168 h. In addition, at 24 and 48 h post-infection, a sample of blood was taken from a tail vein and the CFU/ml was determined by plating serial dilutions.

### Ethics Statement

Mouse experiments at the University of Leicester were performed under appropriate project (permit no. 60/4327) and personal (permit no. 80/10279) licenses according to the United Kingdom Home Office guidelines under the Animals Scientific Procedures Act 1986, and the University of Leicester ethics committee approval. The protocol was approved by both the U.K. Home Office and the University of Leicester ethics committee. Where indicated, the procedures were carried out under anesthesia with isoflurone. Animals were housed in individually ventilated cages in a controlled environment and were frequently monitored after infection. In bacterial infection experiments mice were humanely culled immediately if they became lethargic.

## Results

### TprA Represses *PhrA* Activity in a Sugar Dependent Manner

**Figure 1:**
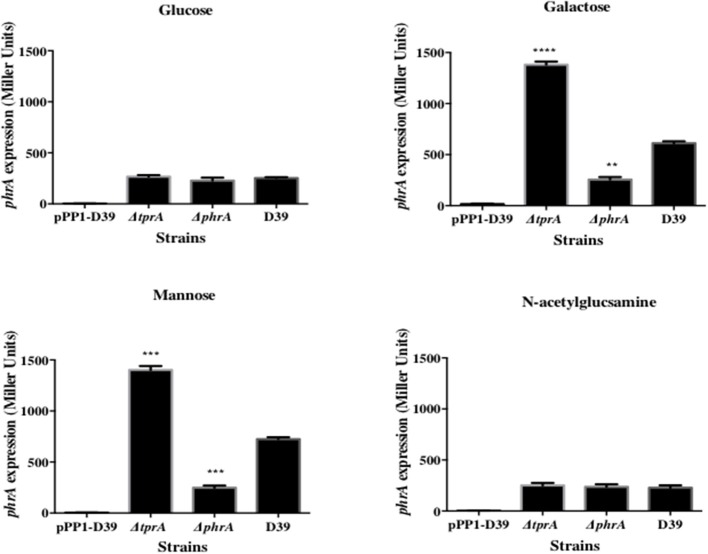
Analysis of *phrA* regulation in different sugars using a LacZ reporter assay. The activity of ß-glactosidase under regulation of P*phrA* is expressed in nmol p-nitrophenol/min/mL (Miller Units). A strain containing promoterless pPP1 plasmid (pPP1-D39) was included as a control in the assay. Values are average of three independent experiments each with three replicates. Bars indicate standard error of mean (SEM). ^**^ *p* < 0.01, ^***^ *p* < 0.001, ^****^ *p* < 0.0001.

The TprA/PhrA system is induced by host-derived sugars, so we studied the influence of TprA on *phrA* in different sugar sources. To this end we utilized a LacZ reporter assay and isogenic mutants. For this, P*tprA* and P*phrA* driven ß-galactosidase activity was measured in wild type or in Δ*tprA* and Δ*phrA*. The results showed that in the condition where the *tprA* promoter activity is high, that is in presence of mannose and galactose, TprA reduces P*phrA*-driven ß-galactosidase activity relative to its expression in the wild type D39 strain, indicating that TprA is a repressor of *phrA* (*p* < 0.01 and *p* < 0.001 in mannose and galactose, respectively) (Figure 1). In glucose or N-acetylglucosamine, where the P*tprA* activity is low, TprA does not have any effect on *phrA* expression (Figure 1). Moreover, it was also found that *phrA* activates its own expression on mannose and galactose, when its promoter activity is up, but this regulatory effect could not be observed on glucose and N-acetylglucosamine (Figure 1).

**Figure 2:**
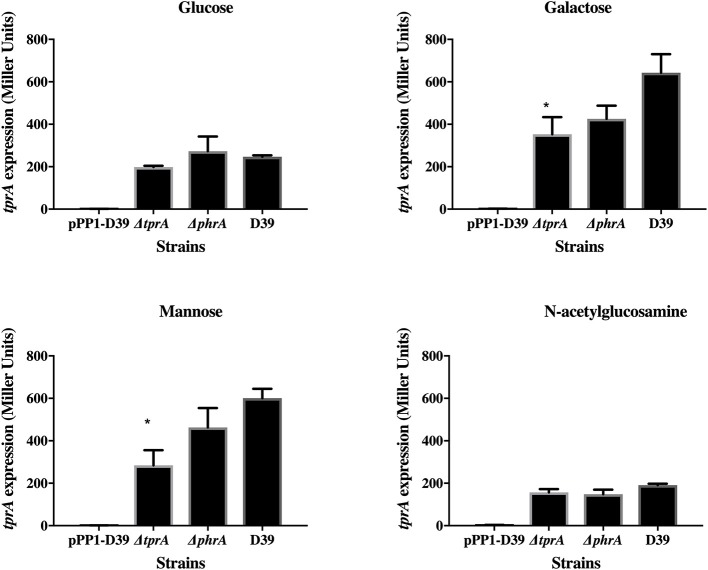
Analysis of *tprA* regulation in different sugars using a LacZ reporter assay. The activity of ß-glactosidase under regulation of P*tprA* is expressed in nmol p-nitrophenol/min/mL (Miller Units). A strain containing promoterless pPP1 plasmid (pPP1-D39) was included as a control in the assay. Values are average of three independent experiments each with three replicates. Bars indicate standard error of mean (SEM). ^*^ *p* < 0.05.

TprA may also positively regulate its own expression (Figure 2). In glucose or N-acetylglucosamine, where TprA levels are low, the absence of *tprA* or *phrA* does not influence *tprA* gene expression levels. In contrast, in galactose and mannose where TprA levels are high, deletion of *tprA* leads to a decrease in *tprA* promoter expression, consistent with autoinduction of TprA. Deletion of *phrA* does not have a significant influence on *tprA* gene expression consistent with TprA also having PhrA-independent activity. Thus, expression of the TprA regulon must depend on the balance between PhrA bound and unbound forms (Figure 2).

### Regulon Determination for TprA on Galactose and Mannose

To analyse the regulon of TprA we selected galactose and mannose growth conditions, where TprA is predicted to be playing an inhibitory role, and where both the TprA promoter and PhrA promoter activities are high. We compared gene expression in wild type and Δ*tprA* mutants using a microarray chip. The results showed that in galactose, 61 genes were differentially expressed, of these 46 were up-regulated, and 15 were down regulated in the mutant (Tables S3–S6). The up-regulated genes could be divided into eight putative operons. The most up-regulated was 11 genes, SPD_1747-SPD_1756, downstream of *phrA*, including a bacteriocin synthesis cluster, that showed between 5- and 73-fold change. This locus is predicted to synthesize lantibiotics and its change in expression in the *tprA* mutant was also reported by Hoover et al. (2015). Lantibiotics are secreted cyclic peptides produced by various bacterial species, and many function as bacteriocins (Willey and van der Donk, 2007). Pneumococci encode a large number of predicted bacteriocins, which have been linked to inter and intra species competitions (Dawid et al., 2007; Rezaei et al., 2018; Wholey et al., 2019). PTS and ABC transporters involved in galactose and mannose uptake, and mannose metabolism were also up-regulated by 2- to 4.5 fold. Additionally, a cluster of genes, SPD_1944-SPD_1948, was up-regulated by 3–8 fold in the mutant. This locus contains a gene for synthesis of a putative CAAX amino terminal protease, for a hypothetical protein, a membrane protein and a putative transcriptional regulator. Previously we demonstrated the expression of this locus was also raised in a mutant of *pflB*, which codes for pyruvate formate lyase, indicating that this locus has a role in control of the mixed acid fermentation seen when the pneumococcus is propagated on galactose or mannose (Al-Bayati et al., 2017). The notably downregulated genes included eight tRNA genes and a GlnR family transcriptional regulator.

On mannose, 29 genes were differentially regulated. Off these 21 were up-regulated and eight were down-regulated. The upregulated genes include three operons: a locus containing putative genes possibly involved in membrane protein synthesis (SPD_1514-SPD_1519), the downstream bacteriocin loci, and the hypothetical set with a CAAX protease. The two latter sets were also observed in the galactose, but the fold changes observed between the presence and absence of TprA, are less accentuated in mannose than galactose. The notably downregulated genes on mannose included SPD_0093, SPD_0094, and SPD_0095. These genes code for hypothetical proteins of unknown function, and are highly conserved in the fully sequenced pneumococcal genomes (Liu et al., 2017). Haas et al. (2005) reported that exposure to a lethal concentration of vancomycin led to significant alteration in the transcription of these genes in multiple *S. pneumoniae* strains.

Direct interaction of TprA with the differentially expressed genes was analyzed by EMSA. To do this, fluorescently labeled DNA probes representing the putative promoters of *phrA* (SPD_1746), a hypothetical protein upregulated in mannose (SPD_1517), and a L-fucose phosphate aldolase upregulated in galactose (SPD_1994) were prepared and mixed with different concentration of recombinant TprA. The result showed that in the presence of TprA, the mobility of labeled probes has changed compared to that of DNA alone, indicative of a direct interaction between TprA and these DNA probes (Figure S1). TprA binding was specific, as the recombinant protein could not bind to the putative promoter of *gyrB*, which is not within TprA regulon.

### CcpA and GlnR Interact With TprA

**Figure 3:**
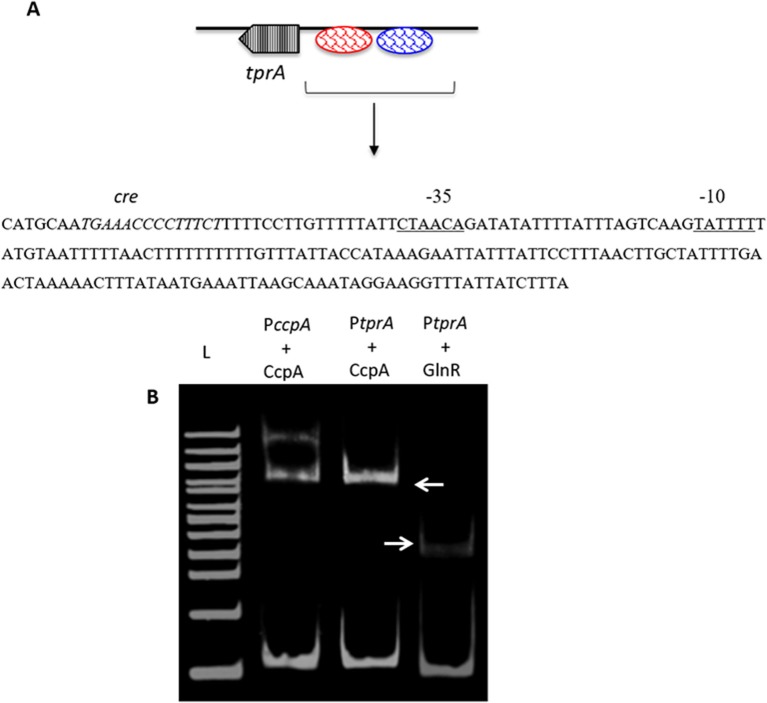
Electrophoretic mobility shift assay showing the direct interaction of His-CcpA and His-GlnR with the putative promoter region of *tprA*. **(A)** Illustration showing the analysis of the predicted promoter region of P*tprA* used in EMSA. The core promoter regions, −10 and −35 sequences, are underlined, and the putative transcriptional factor binding sites are shown in blue, and the putative *cre* sequence is in red. **(B)** Direct interaction of 0.5 μM CcpA and GlnR with the putative promoter sequence of *tprA*. CcpA binding to its own promoter containing *cre* sequence was used as a control (lane L- 500 ng of 100 bp DNA ladder (NEB). Gels were stained with SYBR Green EMSA for visualizing DNA. The arrows indicate the mobility shift.

As TprA/PhrA expression is influenced by different sugars, we reasoned that the system may be controlled by other transcriptional regulators involved in regulation of sugar metabolism, such as CcpA, which is the main transcriptional regulator of carbon catabolite control in *S. pneumoniae* and functions by binding to DNA at catabolite-responsive element (*cre*) sites. Further support for this analysis originated from the fact that a *cre*-like sequence is located in the putative promoter region of *tprA* (Hoover et al., 2015; Figure 3). In addition to CcpA, GlnR's (encoded by SPD_0447) role in regulation of *tprA*, was also investigated as *glnR* was found to be differentially expressed in Δ*tprA* relative to wild type (Table S3). We demonstrated by electrophoretic mobility shift assay (EMSA) analysis that both CcpA and GlnR bind to the putative promoter region of *tprA* (Figure 3), indicating direct interaction of CcpA and GlnR with the putative promoter of *tprA* (P*tprA*). This binding was specific as CcpA and GlnR could not bind to upstream region of *gyrB* (data not shown).

To test the influence of CcpA and GlnR on *tprA* regulation further, we used P*tprA* reporter strains. To this end, P*tprA* was fused to a promoterless *lacZ* and reporter strains were created in wild type (P*tprA*::*lacZ-*wt), Δ*ccpA* (P*tprA*::*lacZ*-Δ*ccpA)*, and Δ*glnR* (P*tprA*::*lacZ*-Δ*glnR*). We found that the P*tprA* activity in P*tprA-lacZ*-Δ*glnR* was lower than in P*tprA*-*lacZ*-wt in both glucose and galactose in the presence or absence of oxygen. This finding suggests that GlnR is a positive regulator of P*tprA* activity and active in multiple sugars and oxygen conditions (Table 1).

**Table 1:** Expression levels (in Miller units) of pneumococcal transcriptional *lacZ*-fusions to the promoter of *tprA* in different genetic backgrounds grown microaerobically and anaerobically in CDM supplemented with 55 mM of glucose or galactose.

**Strains**	**ß-galactosidase activity (Miller units)**			
	**Microaerobic**		**Anaerobic**	
	**Glucose**	**Galactose**	**Glucose**	**Galactose**
pPP1-wt	0.8 ± 0.08	0.7 ± 0.05	0.9 ± 0.07	0.8 ± 0.06
P*tprA*-*lacZ*-wt	19.3 ± 2.3	267.8 ± 4.5	56.0 ± 2.0	275.5 ± 4.4
P*tprA-lacZ*-Δ*ccpA*	135.7 ± 3.2	33.3 ± 1.8	222.6 ± 5.0	119.2 ± 4.6
P*tprA-lacZ*-Δ*glnR*	1.1 ± 0.07	82.1 ± 1.1	1.1 ± 0.07	94.4 ± 8.5

*The activity is expressed as nmol p-nitrophenol/min/mL. Values are average of three independent experiments, each with three replicates. “±” indicates standard error of mean (SEM)*.

On the other hand, P*tprA* activity in P*tprA-lacZ*-Δ*ccpA* was lower than P*tprA*-*lacZ*-wt in galactose, and higher than P*tprA*-*lacZ*-wt in glucose. CcpA is a known repressor, and these findings suggest that it represses P*tprA* expression on glucose and de-represses on galactose (Table 1). These data show that TprA regulation is controlled by multiple regulators, in that regulation via CcpA is highly dependent on carbohydrate sources and promotes *tprA* expression on galactose.

### *In vivo* Studies

**Figure 4:**
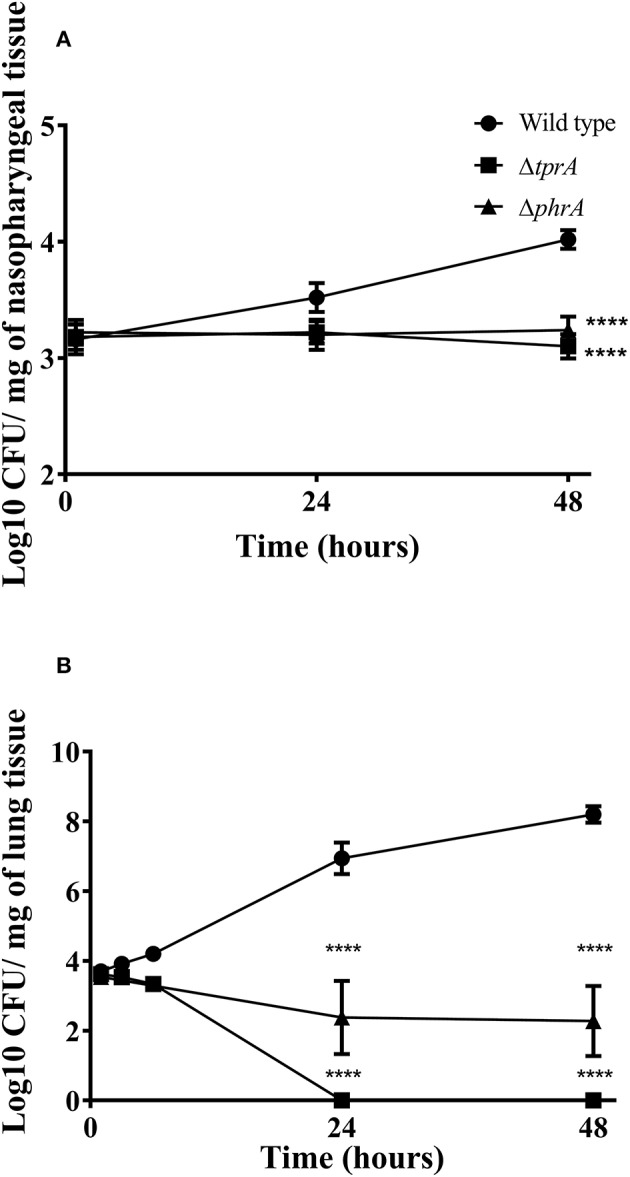
Survival of pneumococcal strains in nasopharynx **(A)** and lungs **(B)**. Mice were infected intranasally with ~1 × 10^6^ CFU/mouse. Each datum point was collected from five animals. Bars indicate standard error of mean (SEM). ^****^p < 0.0001.

Previously, we reported the essential role of TprA/PhrA system in pneumococcal virulence after intranasal infection but did not determine the growth profiles of pneumococcal strains in the nasopharynx, lungs and blood during the course of infection (Motib et al., 2017). In this study, we evaluated TprA/PhrA's role in growth of *S. pneumoniae* in the respiratory tract after intranasal infection, and in blood after intravenous infection by following growth of pneumococci in these tissues. The growth profiles of pneumococcal strains in the nasopharynx and lung, were determined after intranasal infection. In the nasopharynx, the numbers of wild-type D39 bacteria increased gradually by 24 (Log_10_ 3.52 ± 0.12 CFU/mg, *n* = 5) and 48 h (Log_10_ 4.02 ± 0.08 CFU/mg, *n* = 5) post-infection (Figure 4A). In contrast, the numbers of Δ*tprA* and Δ*phrA* did not change at 24 and 48 h post infection (Figure 4A). The difference between the number of bacteria recovered from the wild type and the mutant strains was highly significant (*p* < 0.0001). In the lungs, the numbers of wild type increased approximately two log over 48 h, while the numbers of Δ*phrA* did not change significantly at 24 and 48 h post infection relative to the numbers determined immediately after post-infection (*p* > 0.05). Moreover, the Δ*tprA* was cleared 24 h post infection. These data show that both TprA and PhrA are required for pneumococcal growth in the respiratory tract, however the relative contributions of the individual components of this QS system in pneumococcal survival differ.

The *in vitro* data suggest that TprA/PhrA should not play a key role in after intravenous inoculation, given the higher levels of glucose in the blood. To test this, we compared the wild type, Δ*tprA* and Δ*phrA* in a systemic infection model by administering the strains through the dorsal tail vein. The results showed that all the mice infected either with Δ*tprA* or Δ*phrA* survived the challenge (168 h), whereas five out of six mice infected with D39 reached the assay end point (median survival: 58 ± 48.8 h, *n* = 6). Moreover, the CFU/ml of D39 at 24- and 48 h post infection was Log_10_ 3.25 ± 0.56 (*n* = 6) and Log_10_ 5.95 ± 1.36 (*n* = 6), respectively, whereas Δ*tprA* and Δ*phrA* could not be detected in the blood at these time points. These data demonstrate that the role of the TprA/PhrA extends beyond galactose metabolism contributing to metabolic function in diverse conditions.

### Microarray Analysis of Δ*tprA* on Glucose

In order to explore the reasons behind the growth attenuation of the mutant in blood, we determined, by microarray analysis, the transcriptome of Δ*tprA* on glucose, because the microbe can be exposed to up to 10 mM glucose in blood (Philips et al., 2003). The analysis showed that 53 genes were differentially expressed in Δ*tprA* relative to the wild type: 25 down-regulated and 28 up-regulated in the mutant compared to the wild type, affecting five putative operons, and several other genes with various metabolic functions (Tables S7, S8). In these five putative operons, the expression was up in four affecting the expression of genes involved in the synthesis of dihydroorotate dehydrogenase, endo-beta-N-acetylglucosaminidase (lytB) (SPD_0852-SPD_0853), iron transporters (SPD_0916-SPD_0917), aminotransferase (SPD_0978-SPD_0979), and carbamoyl-phosphate synthase (SPD_1132- SPD_1133), while it was down in one operon affecting a locus responsible for the synthesis of heat shock proteins (SPD_0458-SPD_0460). Other notable differentially expressed genes included transcriptional regulators (*comX1*, SPD_0144, *spxA, hrcA*), the genes encoding for several choline binding proteins including *cbpE* and *pcpA*, and 18 hypothetical genes with unknown function. These data demonstrate that the TprA/PhrA system has a role in wider metabolic functions beyond galactose metabolism.

## Discussion

The mechanism of pneumococcal adaptation in tissues with different sugar composition is not fully understood. We demonstrate that the TprA/PhrA system is one of the regulatory systems involved in metabolism of galactose and mannose, and is essential for pneumococcal survival in tissues. This system can be targeted by linear molecularly imprinted polymers to modulate the consumption of host-derived sugars, the activity of neuraminidase, and pneumococcal survival in the host (Motib et al., 2017).

Previously we demonstrated that the TprA/PhrA system is essential for virulence in a mouse model of pneumonia, as well as in a chinchilla otitis media model (Motib et al., 2017). The results obtained in this study clearly demonstrate that in the absence of this QS system, the pneumococcus cannot grow in the nasopharynx and lungs. The effect of TprA mutation was severe on pneumococcal survival particularly in the lungs because it was cleared off after 24 h post infection. While Δ*phrA* could be detected in the lungs, these pneumococci could not successfully disseminate into the blood (Motib et al., 2017). This shows that TrpA requires PhrA binding for its full function. Growth attenuation of *phrA* mutant is probably due to the lack of activation of TprA/PhrA QS system. However, TprA independent functions of PhrA cannot be ruled out, and this needs to be further investigated. Cross regulatory interaction among the members of RRNPP family are well-documented (Cook and Federle, 2014; Zhi et al., 2018).

The TprA/PhrA system is induced on galactose and mannose, sugars found predominantly in the structure of host glycans in respiratory secretions (King et al., 2006). However, this does not exclude TprA/PhrA system's involvement in other metabolic functions. Both Δ*tprA* and Δ*phrA* were avirulent in septicaemia model, and thus, given that glucose concentration is higher than galactose and mannose in blood, abrogation of virulence is unlikely due only to effects on galactose and mannose metabolism. Thus, we conclude that TprA has functions other than its role in galactose and mannose metabolism. Support for this postulation comes from TprA regulon analysis on these two sugars, which showed that in addition to its involvement in sugar metabolism, TprA controls the expression of genes involved in other pathways including iron metabolism and synthesis of predicted bacteriocins. Our microarray analysis of Δ*tprA* on glucose also supports the view that TprA/PhrA systems regulatory function extends beyond its control over galactose and mannose metabolism.

Attenuation of colonization and virulence of TprA/PhrA mutants in the respiratory tract is very likely due to the inability to cleave, transport and metabolize host sugars. We showed previously by EMSA and by reporter assays that the expression of *nanA*, which encodes for the main pneumococcal neuraminidase activity, is upregulated by TprA on galactose but not on glucose (Motib et al., 2017). The involvement of *nanA* in pneumococcal colonization and virulence has been linked to the removal of terminal sialic acid from host glycoconjugates. The initial cleavage is critical as this process exposes other internal sugar linkages to the activity of pneumococcal glycosidases, such as galactosidases and mannosidases, which cleave galactose and mannose, respectively (King, 2010). The reduction in neuraminidase activity in the Δ*tprA* is consistent with growth attenuation of this mutant on mucin, very likely due to reduced concentration of free sugars and subsequent deprivation of nutrients.

**Figure 5:**
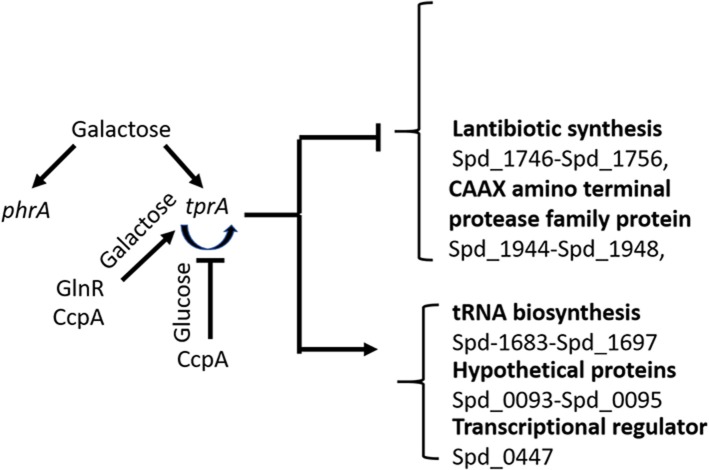
Schematic diagram of TprA/PhrA mediated regulation. Blunt arrows indicate down- and pointed arrows show upregulation. The curve arrows show self-induction. Some of the genes that are up- or down regulated by TprA/PhrA system on mannose and galactose are also shown.

Our results suggest the involvement of the TrpA/PhrA system to the wider metabolic network and show the complexity of regulation of sugar metabolism in *S. pneumoniae* as illustrated in Figure 5. Here we found that *tprA* expression is regulated not only by autoinduction on mannose and galactose, but also by CcpA, the master regulator of sugar metabolism, and by GlnR, a regulator of glutamine and glutamate metabolism (Hendriksen et al., 2008). CcpA is the activator of TprA on galactose and a repressor on glucose. GlnR is an activator independent of sugar type. The presence of multiple regulator-binding sites highlights the intertwined nature of different aspects of central metabolic pathways. It is very likely that these proteins bind to *cre* like sequence located proximal to *tprA*, which is known to be the binding site for CcpA.

Interfering with bacterial communication systems is considered to be an effective strategy to develop novel anti-infectives (Clatworthy et al., 2007; Brooks and Brooks, 2014). QS system homologs are not present in mammals hosts and conserved among pathogenic bacteria, meaning that the possibility of host-toxicity would be low (Clatworthy et al., 2007). We previously demonstrated that TprA/PhrA system is a drugable target. This study adds to our understanding of how this system affects the pneumococcal gene regulation, survival, and virulence.

## Data Availability

The datasets generated for this study can be found in the GEO/GSE 67668.

## Ethics Statement

Mouse experiments at the University of Leicester were performed under appropriate project (permit no. 60/4327) and personal (permit no. 80/10279) licenses according to the United Kingdom Home Office guidelines under the Animals Scientific Procedures Act 1986, and the University of Leicester ethics committee approval. The protocol was approved by both the U.K. Home Office and the University of Leicester ethics committee. Where indicated, the procedures were carried out under anesthesia with isoflurone. Animals were housed in individually ventilated cages in a controlled environment, and were frequently monitored after infection. In bacterial infection experiments mice were humanely culled immediately if they became lethargic.

## Author Contributions

AM, FA-B, IM, SS, AK, OK, NH, PA, and HY made substantial contributions to the conception or design of the work, the acquisition, analysis, and interpretation of data for the work, gave the final approval of the manuscript to be published, and agreed to be accountable for all aspects of the work in ensuring that questions related to the accuracy or integrity of any part of the work are appropriately investigated and resolved. AM, OK, NH, PA, and HY contributed to the drafting the work and critically revising the manuscript for important intellectual content.

### Conflict of Interest Statement

The authors declare that the research was conducted in the absence of any commercial or financial relationships that could be construed as a potential conflict of interest.
