# Supplementary material for: TprA/PhrA Quorum Sensing System Has a Major Effect on Pneumococcal Survival in Respiratory Tract and Blood, and Its Activity Is Controlled by CcpA and GlnR
Source: Front Cell Infect Microbiol. 2019 Sep 13;9:326. doi: 10.3389/fcimb.2019.00326 (PMC6753895; doi:10.3389/fcimb.2019.00326)
Supplement: Supplementary file 1 [file Table_1.DOCX]

**STable 1:** The list of strains and plasmids used in this study.

| Strains/Plasmids | Description/Use | Source |
| --- | --- | --- |
| *S. pneumoniae* |  |  |
| D39 | Serotype 2 strain | Laboratory stock |
| Δ*tprA* | D39; SPD1745:Spec^R^ | This study |
| Δ*tprA*Comp | D39; *tprA* + Δ*tprA*:Spec^R^; Kan^R^ | This study |
| Δ*phrA* | D39; SPD1746:Spec^R^ | This study |
| Δ*phrA*Comp | D39; *phrA*+ Δ*phrA*:Spec^R^; Kan^R^ | This study |
| pPP1::*lacZ*-wt | D39; Δ*bgaA*::pPP1*-lacZ*; Tet^R^ | This study |
| P*ccpA*::*lacZ*-wt | D39; Δ*bgaA*::P*ccpA-lacZ*; Tet^R^ | Al-Bayati et al., 2017 |
| P*tprA*::*lacZ*-wt | D39; Δ*bgaA*::P*tprA-lacZ*; Tet^R^ | This study |
| P*merR*^2^::*lacZ*-wt | D39; Δ*bgaA*::P*merR^2^-lacZ*; Tet^R^ | Al-Bayati et al., 2017 |
| P*phrA*::*lacZ*-wt | D39; Δ*bgaA*::P*phrA-lacZ*; Tet^R^ | This study |
| P*tprA::lacZ*-Δ*tprA* | Δ*tprA*:Spec^R^; Δ*bgaA*::P*tprA-lacZ*; Tet^R^ | This study |
| P*tprA::lacZ*-Δ*phrA* | Δ*phrA*:Spec^R^; Δ*bgaA*::P*tprA-lacZ*; Tet^R^ | This study |
| P*phrA::lacZ*-Δ*phrA* | Δ*phrA*:Spec^R^; Δ*bgaA*::P*phrA-lacZ*; Tet^R^ | This study |
| P*phrA::lacZ*-Δ*tprA* | Δ*tprA*:Spec^R^;Δ*bgaA*::P*phrA-lacZ*; Tet^R^ | This study |
| *Escherichia coli* |  |  |
| One Shot® TOP10 | Plasmid propagation | Invitrogen, UK |
| BL21 (DE3) pLysS | Protein expression | Agilent Tech, USA |
| Plasmids |  |  |
| pDL278 | Amplification of Spec^R^ (*aadA*) | (Yesilkaya, 1999) |
| pLEICS-01 | 6His-Tag for protein expression; Amp^R^ | PROTEX, UK |
| pCEP | Genetic complementation; Kan^R^ | (Guiral et al., 2006) |
| pPP1 | Promoterless *lacZ* for transcriptional fusions; Amp^R^ Tet^R^ | (Halfmann et al., 2007) |
