# Supplementary material for: TprA/PhrA Quorum Sensing System Has a Major Effect on Pneumococcal Survival in Respiratory Tract and Blood, and Its Activity Is Controlled by CcpA and GlnR
Source: Front Cell Infect Microbiol. 2019 Sep 13;9:326. doi: 10.3389/fcimb.2019.00326 (PMC6753895; doi:10.3389/fcimb.2019.00326)
Supplement: Supplementary file 2 [file Table_2.DOCX]

**STable 2.** The list of primers used in this study.

| **Primer ID** | **Sequence (5’- 3’)** |
| --- | --- |
| SOEing PCR primers |  |
| Spec-F | ATCGATTTTCGTTCGTGAATACATGTTAT |
| Spec-R | GTTATGCAAGGGTTTATTGTTTTCTA |
| LF-SOE0447-F | TCGATAAATCCCAGTTCAAACTT |
| LF-SOE0447-R | TATTCACGAACGAAAATCGATGCGAAATTCTTTTTCCTTCATTT |
| RF-SOE0447-F | AACAATAAACCCTTGCATAACTCACCTTTTGGTCGCGGTTAGGC |
| RF-SOE0447-R | TCCCATTTTGGTCAAGACATTCA |
| LF-SOE1745-F | GCGTCCAACGTGGCTCTGCACCA |
| LF-SOE1745-R | TATTCACGAACGAAAATCGATTTTCTCTGCGAGTGTATTCATTA |
| RF-SOE1745-F | AACAATAAACCCTTGCATAACAAGGAACTAGATACTGTTTAGTT |
| RF-SOE1745-R | GCTAGGACACTATGGACTTCTTG |
| LF-SOE1746-F | TTTCCAATGACGAAATTGCCT |
| LF-SOE1746-R | CCTCTCTCAAAATTATTAGTT |
| RF-SOE1746-F | ACGGATCCCCAGCATGCGCGCAACTAATTTTTTAATTCCACG |
| RF-SOE1746-R | TTACTGGATGAATTGTTTTAGAATGGTCTTGATGTTGGGAAG |
| LF-SOE1797K-F | GAATCGCCCGGGGCTTATCCAAC |
| LF-SOE1797K-R | TGCGCGCCATGGTAGCACCTCTACTGTATCATCTGCATTCATTC |
| RF-SOE1797K-F | TTACTGGATGAATTGTTTTAGTCAACACGAAAACGTAAATAGAA |
| RF-SOE1797K-R | TATGACAGATGAGCTGATTGATA |
| Complementation primers |  |
| SPD1745-Comp-F | CGCCATGGGTAATTTTTAACTTTTTTTT |
| SPD1745-Comp-R | CGGGATCCCTAAACAGTATCTAGTTCCT |
| MalF | GCTTGAAAAGGAGTATACTT |
| pCEPR | AGGAGACATTCCTTCCGTATC |
| SPD0447-C-F | TACTTCCAATCCATGAAGGAAAAAGAATTTCGCCGA |
| SPD0447-C-R | TATCCACCTTTACTGTCAACCGCGACCAAAAGGTGA |
| SPD1797-C-F | TACTTCCAATCCATGAATGCAGATGATACAGTAA |
| SPD1797-C-R | TATCCACCTTTACTGTCATTTACGTTTTCGTGTTGA |
| EMSA primers |  |
| PccpA-EMSA-F | TGAAATTTTTTCAATCAATCTTCA |
| PccpA-EMSA-R | GTGAAAATTTCGTTTTCATATTT |
| PmerR-EMSA-F | CATTATCAATTGACGTTTGT |
| PmerR-EMSA-F | CCATATTTCGGCGAAATTCT |
| Fusion primers |  |
| PtprA-Fusion-F | CGGCATGCCTTTCTTTTTCCTTGTTTTT |
| PtprA-Fusion-R | CGGGATCCATCTGAATTTCTCTGCGAGT |
| PphrA-Fusion-F | CGGCATGCTAAAGATAATAAACCTTCCT |
| PphrA-Fusion-R | CGGGATCCATTTTTTAATTCCACGTTTT |
| PnanA- Fusion-F | CGGCATGCGCAGGAAGTATGGTGTAAAT |
| PnanA- Fusion-R | CGGGATCCCGTTCCAAATACCACTGCTC |
