# Supplementary material for: TprA/PhrA Quorum Sensing System Has a Major Effect on Pneumococcal Survival in Respiratory Tract and Blood, and Its Activity Is Controlled by CcpA and GlnR
Source: Front Cell Infect Microbiol. 2019 Sep 13;9:326. doi: 10.3389/fcimb.2019.00326 (PMC6753895; doi:10.3389/fcimb.2019.00326)
Supplement: Supplementary file 3 [file Table_3.DOCX]

**STable 3:** Summary of transcriptome comparison of *S. pneumoniae* D39 Δ*tprA* and wild-type, grown in CDM plus galactose (Downregulated genes in Δ*tprA*)

| **Gene tag^a^** | **Function^b^** | **Ratio^c^** | **P-value** |
| --- | --- | --- | --- |
| spd_0015 |  | -2.07 | 1.05E-02 |
| spd_0447 | Transcriptional regulator, MerR family protein | -2.02 | 8.18E-03 |
| spd_0756 |  | -3.66 | 4.20E-04 |
| spd_0913 | Hypothetical protein | -1.99 | 8.31E-03 |
| spd_0936 | Tn5252, relaxase | -2.06 | 9.16E-02 |
| spd_1410 |  | -4.81 | 7.12E-04 |
| spd_1683 |  | -3.1 | 9.87E-03 |
| spd_1685 |  | -2.01 | 1.48E-02 |
| spd_1687 |  | -2.62 | 2.46E-03 |
| spd_1694 |  | -3.37 | 7.31E-04 |
| spd_1695 |  | -2.44 | 6.89E-03 |
| spd_1696 |  | -3.02 | 1.79E-03 |
| spd_1697 |  | -2.08 | 1.31E-02 |
| spd_1745 | Transcriptional regulator PlcR, putative | -3.08 | 7.85E-04 |
| spd_1882 |  | -3.16 | 1.67E-03 |

^a^Gene numbers refer to D39 locus tags. ^b^D39 annotation. ( Lanie et al., 2007), ^c^Ratios >2.0 or <2.0.
