# Supplementary material for: TprA/PhrA Quorum Sensing System Has a Major Effect on Pneumococcal Survival in Respiratory Tract and Blood, and Its Activity Is Controlled by CcpA and GlnR
Source: Front Cell Infect Microbiol. 2019 Sep 13;9:326. doi: 10.3389/fcimb.2019.00326 (PMC6753895; doi:10.3389/fcimb.2019.00326)
Supplement: Supplementary file 4 [file Table_4.DOCX]

**STable 4**: Summary of transcriptome comparison of *S. pneumoniae* D39 Δ*tprA* and wild-type grown in CDM plus galactose. (Upregulated genes in Δ*tprA*).

| **Gene tag^a^** | **Function^b^** | **Ratio^c^** | **P-value** |
| --- | --- | --- | --- |
| Spd_0008 | Septum formation initiator, putative | 2.96 | 7.27E-03 |
| Spd_0010 | Hypothetical protein | 2.66 | 1.12E-02 |
| Spd_0120 | Hypothetical protein | 2.14 | 2.46E-02 |
| Spd_0323 | Polysaccharide polymerase | 2.33 | 1.57E-02 |
| Spd_0325 | Hypothetical protein | 2.18 | 2.09E-02 |
| Spd_0453 | Type I restriction-modification system, S subunit | 3.29 | 2.36E-03 |
| Spd_0558 | Cell wall-associated serine protease PrtA | 2.53 | 4.03E-02 |
| Spd_0559 | PTS system IIA component, putative | 2.34 | 5.86E-03 |
| Spd_0560 | PTS system, IIB component, putative | 2.91 | 7.13E-04 |
| Spd_0750 | Hypothetical protein | 2.16 | 1.64E-02 |
| Spd_0915 | Iron-compound ABC transporter, iron compound-binding protein | 2.56 | 3.87E-03 |
| Spd_0920 | Hypothetical protein | 2.12 | 7.90E-03 |
| Spd_0932 | Hypothetical protein | 2.16 | 6.32E-03 |
| Spd_1057 | PTS system, IIB component, putative | 2.76 | 1.28E-03 |
| Spd_1058 |  | 2.52 | 2.69E-03 |
| Spd_1167 | ABC transporter, ATP-binding protein | 2.86 | 5.02E-03 |
| Spd_1169 | Oligopeptide ABC transporter, permease protein | 2.24 | 6.89E-03 |
| Spd_1172 | N-acetylmannosamine-6-phosphate 2-epimerase 2, putative | 3.39 | 6.87E-04 |
| Spd_1495 | Sugar ABC transporter, sugar-binding protein | 3.94 | 3.87E-04 |
| Spd_1496 | PTS system, IIBC components | 4.55 | 1.98E-04 |
| Spd_1497 | N-acetylmannosamine-6-phosphate 2-epimerase 2, putative | 3.61 | 4.39E-04 |
| Spd_1595 | Hypothetical protein | 2.37 | 4.52E-03 |
| Spd_1746 | Hypothetical protein | 73.25 | 2.15E-07 |
| Spd_1747 | Hypothetical protein | 7.95 | 9.39E-05 |
| Spd_1748 | Hypothetical protein | 15.1 | 1.20E-05 |
| Spd_1749 | Bacteriocin formation protein, putative | 13.08 | 1.75E-05 |
| Spd_1750 | Multimeric flavodoxin WrbA (general function prediction only) | 14.38 | 8.33E-06 |
| Spd_1751 | Hypothetical protein | 49.43 | 7.00E-07 |
| Spd_1752 | Toxin secretion ABC transporter, ATP-binding/permease protein | 5.05 | 1.15E-04 |
| Spd_1753 | Serine protease, subtilase family protein | 25.8 | 2.98E-06 |
| Spd_1754 | Hypothetical protein | 14.06 | 3.05E-06 |
| Spd_1755 | ABC transporter, ATP-binding protein | 7.3 | 1.99E-05 |
| Spd_1756 | Hypothetical protein | 10.2 | 5.37E-06 |
| Spd_1784 | ABC transporter, ATP-binding protein | 2.4 | 3.26E-03 |
| Spd_1944 | CAAX amino terminal protease family protein | 4.24 | 1.83E-04 |
| Spd_1945 | Hypothetical protein | 3.67 | 2.68E-04 |
| Spd_1946 | Hypothetical protein | 6.4 | 3.35E-05 |
| Spd_1947 | Transcriptional regulator, putative | 8.41 | 1.93E-05 |
| Spd_1948 | Hypothetical protein | 7.73 | 1.39E-05 |
| Spd_1989 | PTS system, IID component | 2.84 | 3.16E-03 |
| Spd_1990 | PTS system, IIC component | 3.86 | 7.10E-04 |
| Spd_1991 | PTS system, IIB component | 4.06 | 9.23E-04 |
| Spd_1992 | PTS system, IIA component | 3.03 | 3.70E-03 |
| Spd_1993 | RbsD/FucU transport protein family protein | 3.39 | 2.68E-03 |
| Spd_1994 | L-fuculose phosphate aldolase | 2.4 | 8.59E-03 |
| Spd_2033 | Ribosomal subunit interface protein | 2.37 | 7.57E-03 |

^a^Gene numbers refer to D39 locus tags. ^b^D39 annotation. ( Lanie et al., 2007), ^c^Ratios >2.0 or <2.0 galactose.
