# Supplementary material for: TprA/PhrA Quorum Sensing System Has a Major Effect on Pneumococcal Survival in Respiratory Tract and Blood, and Its Activity Is Controlled by CcpA and GlnR
Source: Front Cell Infect Microbiol. 2019 Sep 13;9:326. doi: 10.3389/fcimb.2019.00326 (PMC6753895; doi:10.3389/fcimb.2019.00326)
Supplement: Supplementary file 6 [file Table_6.DOCX]

**STable 6:** Summary of transcriptome comparison of *S. pneumoniae* D39 Δ*tprA* and wild-type grown in CDM plus mannose (Upregulated genes in Δ*tprA*).

| **Gene tag^a^** | **Function^b^** | **Ratio^c^** | **P-value** |
| --- | --- | --- | --- |
| Spd_0558 | Cell wall-associated serine protease PrtA | 2.4 | 5.60E-03 |
| Spd_1513 |  | 3.72 | 5.35E-05 |
| Spd_1514 | ABC transporter, ATP-binding protein | 4.93 | 5.69E-04 |
| Spd_1515 | Hypothetical protein | 2.62 | 1.55E-02 |
| Spd_1516 | Hypothetical protein | 4.04 | 2.90E-03 |
| Spd_1517 | Hypothetical protein | 2.93 | 2.75E-02 |
| Spd_1746 | Hypothetical protein | 8.7 | 1.65E-07 |
| Spd_1747 | Hypothetical protein | 7.37 | 7.77E-06 |
| Spd_1748 | Hypothetical protein | 9.49 | 4.35E-07 |
| Spd_1749 | Bacteriocin formation protein, putative | 4.39 | 1.55E-05 |
| Spd_1750 | Bultimeric flavodoxin WrbA (general function prediction only) | 3.4 | 5.61E-05 |
| Spd_1751 | Hypothetical protein | 9.49 | 5.96E-08 |
| Spd_1753 | Serine protease, subtilase family protein | 7.5 | 7.58E-08 |
| Spd_1754 | Hypothetical protein | 4.52 | 2.21E-06 |
| Spd_1755 | ABC transporter, ATP-binding protein | 3.08 | 8.26E-05 |
| Spd_1756 | Hypothetical protein | 3.95 | 6.47E-06 |
| Spd_1944 | CAAX amino terminal protease family protein | 3.72 | 5.70E-05 |
| Spd_1945 | Hypothetical protein | 2.04 | 2.96E-02 |
| Spd_1946 | Hypothetical protein | 4.71 | 9.12E-06 |
| Spd_1947 | Transcriptional regulator, putative | 3.92 | 8.85E-05 |
| Spd_1948 | Hypothetical protein | 4.21 | 9.46E-06 |

^a^Gene numbers refer to D39 locus tags. ^b^D39 annotation. ( Lanie et al., 2007), ^c^Ratios >2.0 or <2.0.
