# Supplementary material for: TprA/PhrA Quorum Sensing System Has a Major Effect on Pneumococcal Survival in Respiratory Tract and Blood, and Its Activity Is Controlled by CcpA and GlnR
Source: Front Cell Infect Microbiol. 2019 Sep 13;9:326. doi: 10.3389/fcimb.2019.00326 (PMC6753895; doi:10.3389/fcimb.2019.00326)
Supplement: Supplementary file 7 [file Table_7.DOCX]

**STable 7:** Summary of transcriptome comparison of *S. pneumoniae* D39 Δ*tprA* and wild-type grown in CDM plus glucose (Upregulated genes in Δ*tprA*).

| **Gene tag^a^** | **Function^b^** | **Ratio^c^** | **P-value** |
| --- | --- | --- | --- |
| SPD_0012 | hypoxanthine-guanine phosphoribosyltransferase | -2.39 | 3.53E-10 |
| SPD_0144 | transcriptional regulator | -1.63 | 1.74E-08 |
| SPD_0178 | transcriptional regulator Spx | -1.87 | 2.75E-10 |
| SPD_0401 | 50S ribosomal protein L28 | -1.94 | 4.90E-06 |
| SPD_0437 | hypothetical protein SPD_0437 | -2.26 | 3.75E-08 |
| SPD_0458 | heat-inducible transcription repressor | -2.06 | 8.83E-11 |
| SPD_0460 | molecular chaperone DnaK | -1.61 | 9.10E-09 |
| SPD_0490 | hypothetical protein SPD_0490 | -1.7 | 1.92E-06 |
| SPD_0507 | hypothetical protein SPD_0507 | -1.65 | 2.60E-05 |
| SPD_0527 | oxidoreductase | -1.73 | 8.22E-09 |
| SPD_0564 | hypothetical protein SPD_0564 | -1.63 | 2.36E-05 |
| SPD_0703 | hypothetical protein SPD_0703 | -1.68 | 6.91E-07 |
| SPD_0754 | hypothetical protein SPD_0754 | -1.68 | 1.51E-08 |
| SPD_0809 | lysine decarboxylase | -1.63 | 1.15E-08 |
| SPD_0904 | thymidine kinase | -1.68 | 3.21E-10 |
| SPD_1041 | glutaredoxin-like protein NrdH | -1.79 | 1.87E-07 |
| SPD_1148 | 50S ribosomal protein L19 | -2.02 | 3.98E-11 |
| SPD_1236 | transcriptional regulator Spx | -2.24 | 1.05E-09 |
| SPD_1413 | ATP-dependent RNA helicase | -1.71 | 2.67E-09 |
| SPD_1439 | 30S ribosomal protein S15 | -2.06 | 2.15E-10 |
| SPD_1665 | trehalose operon repressor | -1.88 | 8.11E-09 |
| SPD_1745 | transcriptional regulator PlcR | -2.26 | 1.58E-10 |
| SPD_1746 | hypothetical protein SPD_1746 | -1.61 | 3.13E-03 |
| SPD_1864 | hypothetical protein SPD_1864 | 1.99 | 5.95E-10 |
| SPD_1943 | hypothetical protein SPD_1943 | 1.67 | 1.07E-08 |

^a^Gene numbers refer to D39 locus tags. ^b^D39 annotation. (Lanie et al., 2007). ^c^Ratios >1.5 or <1.5.
