# Supplementary material for: TprA/PhrA Quorum Sensing System Has a Major Effect on Pneumococcal Survival in Respiratory Tract and Blood, and Its Activity Is Controlled by CcpA and GlnR
Source: Front Cell Infect Microbiol. 2019 Sep 13;9:326. doi: 10.3389/fcimb.2019.00326 (PMC6753895; doi:10.3389/fcimb.2019.00326)
Supplement: Supplementary file 8 [file Table_8.DOCX]

**STable 8:** Summary of transcriptome comparison of *S. pneumoniae* D39 Δ*tprA* and wild-type grown in CDM plus glucose (Upregulated genes in Δ*tprA*).

| **Gene tag^a^** | **Function^b^** | **Ratio^c^** | **P-value** |
| --- | --- | --- | --- |
| SPD_0014 | transcriptional regulator ComX1 | 1.89 | 1.88E-09 |
| SPD_0094 | hypothetical protein SPD_0094 | 1.6 | 1.03E-07 |
| SPD_0382 | trans-2-enoyl-ACP reductase II | 1.65 | 9.75E-10 |
| SPD_0394 | hypothetical protein SPD_0394 | 1.7 | 1.02E-08 |
| SPD_0536 | beta-lactam resistance factor | 1.74 | 2.43E-08 |
| SPD_0542 | dipeptidase PepV | 1.68 | 3.65E-09 |
| SPD_0660 | cell division ABC transporter permease FtsX | 1.62 | 7.51E-08 |
| SPD_0821 | choline binding protein E | 1.67 | 1.53E-07 |
| SPD_0836 | hypothetical protein SPD_0836 | 1.76 | 8.17E-08 |
| SPD_0852 | dihydroorotate dehydrogenase 1B | 1.99 | 8.82E-11 |
| SPD_0853 | endo-beta-N-acetylglucosaminidase precursor | 1.69 | 8.08E-09 |
| SPD_0916 | iron-compound ABC transporter permease | 1.69 | 1.65E-07 |
| SPD_0917 | iron-compound ABC transporter permease | 1.87 | 4.32E-08 |
| SPD_0931 | hypothetical protein SPD_0931 | 1.7 | 5.81E-07 |
| SPD_0959 | hypothetical protein SPD_0959 | 2.14 | 3.63E-10 |
| SPD_0978 | hypothetical protein SPD_0978 | 1.77 | 9.95E-06 |
| SPD_0979 | aminotransferase, class V | 1.78 | 6.30E-08 |
| SPD_1062 | DNA repair protein RecN | 1.6 | 1.36E-08 |
| SPD_1132 | carbamoyl phosphate synthase small subunit | 2.11 | 2.72E-10 |
| SPD_1133 | aspartate carbamoyltransferase catalytic subunit | 1.62 | 1.14E-08 |
| SPD_1296 | glutamine amidotransferase subunit PdxT | 1.73 | 3.05E-08 |
| SPD_1366 | hypothetical protein SPD_1366 | 1.91 | 5.49E-10 |
| SPD_1403 | 1,4-beta-N-acetylmuramidase, | 1.77 | 8.29E-10 |
| SPD_1527 | hypothetical protein SPD_1527 | 1.65 | 1.91E-07 |
| SPD_1652 | iron-compound ABC transporter iron-compound-binding protein | 1.88 | 3.44E-08 |
| SPD_1848 | hypothetical protein SPD_1848 | 2.08 | 9.68E-10 |
| SPD_1965 | choline binding protein PcpA | 1.77 | 3.22E-05 |
| SPD_2024 | ABC transporter ATP-binding protein | 1.76 | 2.18E-09 |

^a^Gene numbers refer to D39 locus tags. ^b^D39 annotation. (Lanie et al., 2007). ^c^Ratios >1.5 or <1.5
