# Supplementary material for: TprA/PhrA Quorum Sensing System Has a Major Effect on Pneumococcal Survival in Respiratory Tract and Blood, and Its Activity Is Controlled by CcpA and GlnR
Source: Front Cell Infect Microbiol. 2019 Sep 13;9:326. doi: 10.3389/fcimb.2019.00326 (PMC6753895; doi:10.3389/fcimb.2019.00326)
Supplement: Supplementary file 9 [file Data_Sheet_1.docx]

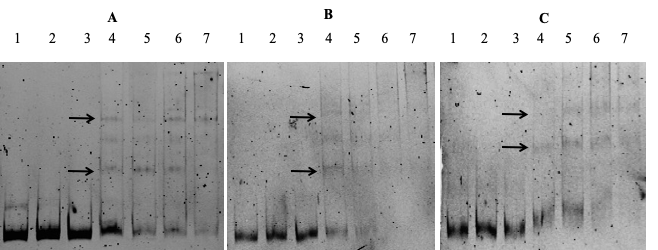


**SFigure 1: Direct interaction of recombinant TprA with the putative promoters of SPD_1746 (A), SPD_1517 (B) and SPD_1994 (C) by EMSA.** In each panel lane 1 indicates the labelled probes representing the upstream region of *gyrB* (10 nmol); Lane 2, *gyrB* probe with 0.5 μM recombinant TprA (negative control); Lane 3, labelled 10 nmol probe alone representing the putative promoters of SPD_1746 (A), SPD_1517 (B) or SPD_1994 (C), while lanes 4-7 represent the binding reactions containing the labelled respective probes and different concentration (0.2-0.5 nM) of recombinant TprA. Positive protein-DNA interactions are seen in lanes 4-7 and indicated with arrows.
