# Supplementary material for: TprA/PhrA Quorum Sensing System Has a Major Effect on Pneumococcal Survival in Respiratory Tract and Blood, and Its Activity Is Controlled by CcpA and GlnR
Source: Front Cell Infect Microbiol. 2019 Sep 13;9:326. doi: 10.3389/fcimb.2019.00326 (PMC6753895; doi:10.3389/fcimb.2019.00326)
Supplement: Supplementary file 10 [file Data_Sheet_2.docx]

**Supplementary References**

Al-Bayati F.A., Kahya HF, Damianou A, Shafeeq S, Kuipers OP, Andrew PW, et al. (2017). Pneumococcal galactose catabolism is controlled by multiple regulators acting on pyruvate formate lyase*. Sci Rep*. 27, 43587. doi: 10.1038/srep43587.

Guiral, S., Hénard, V., Laaberki, M.-H., Granadel, C., Prudhomme, M., Martin, B., et al. (2006). Construction and evaluation of a chromosomal expression platform (CEP) for ectopic, maltose-driven gene expression in *Streptococcus pneumoniae*. *Microbiology* 152, 343–9. doi:10.1099/mic.0.28433-0.

Halfmann, A., Hakenbeck, R., and Brückner, R. (2007). A new integrative reporter plasmid for *Streptococcus pneumoniae*. *FEMS Microbiol. Lett.* 268, 217–24. doi:10.1111/j.1574-6968.2006.00584.x.

Lanie, J. A., Ng, W. L., Kazmierczak, K. M., Andrzejewski, T. M., Davidsen, T. M., Wayne, K. J., et al. (2007). Genome sequence of Avery’s virulent serotype 2 strain D39 of Streptococcus pneumoniae and comparison with that of unencapsulated laboratory strain R6. *J. Bacteriol.* 189, 38–51. doi:10.1128/JB.01148-06.

Yesilkaya, H. (1999). *Studies on the role of superoxide dismutase (SOD) in the virulence of* Streptococcus pneumoniae *and the effects of interferon gamma on sensitivity of phagocytes to the toxin pneumolysin. PhD Thesis*. UK: University of Leicester.
